# Aging, Spatial Disparity, and the Sound-Induced Flash Illusion

**DOI:** 10.1371/journal.pone.0143773

**Published:** 2015-11-30

**Authors:** Denton J. DeLoss, George J. Andersen

**Affiliations:** Department of Psychology, University of California Riverside, Riverside, California, United States of America; Ecole Polytechnique Federale de Lausanne, SWITZERLAND

## Abstract

The present study examined age-related differences in multisensory integration and the effect of spatial disparity on the sound-induced flash illusion—-an illusion used in previous research to assess age-related differences in multisensory integration. Prior to participation in the study, both younger and older participants demonstrated their ability to detect 1–2 visual flashes and 1–2 auditory beep presented unimodally. After passing the pre-test, participants were then presented 1–2 flashes paired with 0–2 beeps that originated from one of five speakers positioned equidistantly 100cm from the participant. One speaker was positioned directly below the screen, two speakers were positioned 50cm to the left and right from the center of the screen, and two more speakers positioned to the left and right 100cm from the center of the screen. Participants were told to report the number of flashes presented and to ignore the beeps. Both age groups showed a significant effect of the beeps on the perceived number of flashes. However, neither younger nor older individuals showed any significant effect of spatial disparity on the sound-induced flash illusion. The presence of a congruent number of beeps increased accuracy for both older and younger individuals. Reaction time data was also analyzed. As expected, older individuals showed significantly longer reaction times when compared to younger individuals. In addition, both older and younger individuals showed a significant increase in reaction time for fusion trials, where two flashes and one beep are perceived as a single flash, as compared to congruent single flash trials. This increase in reaction time was not found for fission trials, where one flash and two beeps were perceived as two flashes. This suggests that processing may differ for the two forms for fission as compared to fusion illusions.

## Introduction

Our senses were once believed to be modular and encapsulated, with information from one sensory modality being inaccessible to the other senses. Any integration of information across the senses was thought to occur in specific “polysensory” areas of the brain [1,2]. However, recent research has shown that this is incorrect with evidence that sensory processing is primarily multisensory in nature [3]. For example, direct connections from auditory cortex to primary visual cortex, and connections from somatosensory cortex to auditory cortex have been identified [4,5]. These findings suggest that unisensory processing that is isolated from other sensory modalities is uncommon. Given that sensory connections across multiple modalities have been found, research on how the senses interact is important in understanding the basic properties of sensory processing.

In the present study we examined age-related differences in multisensory processing. It is well documented in the literature that older individuals are prone to a wide range of sensory declines that occur as a result of normal aging. These declines have a significant influence on the aging population’s health and quality of life [6,7]. Given these sensory declines in older individuals, an important issue is whether declines also occur for multisensory processing.

Recent research has shown that older individuals demonstrate a wide range of changes in processing multisensory stimuli. For example, older individuals have a reduced ability to inhibit irrelevant cross-modal information in multisensory tasks. Older individuals have also demonstrated increased multisensory integration [8–11]. In addition, older adults, as compared to younger adults, exhibit greater improvements in reaction times for multisensory as compared to unisensory stimuli in simple detection tasks.

In contrast, other studies have found a decrease in integration in older individuals [12,13]. One of these studies examined the bounce-stream illusion [12]. Consider two visual objects (discs) approaching each other that overlap and pass by one another. The stimuli can be perceived as either bouncing off one another or streaming through each other. However, when accompanied by a sharp sound as the discs begin to overlap the percept is biased towards perceiving a bouncing of the objects off one another. The second study [13] examined aging and multisensory reaction time facilitation based on distance that involved a soccer ball presented with a 1000Hz tone that was 6dB louder to specify a near as compared to far location. Neither study found evidence of integration for older adults. However, it is worth noting that both studies require additional contextual processing of the stimuli (e.g. bouncing or streaming; near or far) that is not present in other studies of aging and multisensory integration.

Age-related differences in multisensory integration may be evidence of a compensatory mechanism that increases integration in an effort to help counteract age-related declines in vision and audition. However, given the wide range of declines in vision and audition, it seems unlikely that multisensory processing would be spared. Another possibility is that these changes in integration in older individuals reflect a decline in multisensory processing. Multisensory integration is dependent on spatial and temporal coherence, as spatial or temporal disparity increases the degree of integration decreases [14]. In multisensory neurons, the response to spatially or temporally disparate stimuli can even be weaker than its unisensory response to either stimulus modality alone, this is typically referred to as response depression [15]. If integration were to continue for large spatial and temporal disparities, the system would be highly inefficient and the likelihood of integrating information from separate events in the environment would cause considerable increases in erroneous integration. Previous research has shown that older individuals have larger temporal windows of integration [11,16]. Given increased temporal windows of integration, older individuals may also suffer from declines in the efficient spatial integration of multisensory information and demonstrate larger spatial fields of integration. However, to our knowledge, no study has examined whether older individuals show integration across larger spatial disparities as compared to younger individuals.

In the present study we examined age-related declines in spatial coherence of multisensory stimuli using the sound-induced flash illusion. This illusion occurs when a discrepant number of beeps are paired with a number of visual flashes. The illusion occurs when the beeps influence the perceived number of flashes [17,18]. The illusion can be further evaluated in two distinct misperceptions. The first misperception is the fission illusion, when a single flash is paired with two beeps and is perceived as two flashes. The second misperception is the fusion illusion when two flashes are paired with a single beep and perceived as a single flash. The sound induced flash illusion has been used to show that older individuals demonstrate greater integration than younger individuals [8]. It has also been used to show that older individuals have larger temporal windows of integration, and that larger temporal windows of integration are associated with a greater risk of falls in older individuals [16,19]. However, to date, no study has examined age-related differences in the sound-induced flash illusion when the spatial disparity of the stimuli is varied.

To our knowledge, only a single study has examined the role of spatial disparity in the sound induced flash illusion with young adults [20]. This study assessed the role of spatial disparity on the illusion in young adults by presenting the flashes at one of two locations, offset to the left and right of the center of the display by 10 degrees. Speakers were placed directly below the two possible flash locations, allowing for a maximum spatial disparity of 20 degrees. The study found no effect of spatial disparity on the illusion. However, spatial disparity has been found to influence multisensory integration in a number of human and animal studies [21–24]. These studies found an effect of spatial disparity on multisensory integration and saccades [21], event related potentials [23], and neuronal activation of cells in superior colliculus [22] and auditory cortex [24]. Given this extensive literature demonstrating an effect of spatial disparity on multisensory integration, we examined the effect of spatial disparity on the sound induced flash illusion, and examined how this effect might vary with age. Studies that examined spatial disparity and multisensory integration examined disparities greater than that used in the study of spatial disparity and the sound induced flash illusion. As a result, the present study examined the effect of spatial disparity on the sound induced flash illusion with spatial disparities larger than that used in previous research [20]. Specifically, the largest spatial disparity in the previous study was 20 deg. To examine whether spatial disparity influences the sound-induced flash illusion the horizontal disparities used in this study were greater (up to 50 deg) than the disparity used in previous research. Reaction times were also recorded to examine whether any differences in processing occur with increased spatial disparity for older as compared to younger individuals.

## Methods

### Participants

Twelve college students, 6 male and 6 female (*M age* = 23.17 years, *SD* = 4.26 years), from the University of California, Riverside and twelve older participants, 6 male and 6 female (*M age* = 71.45 years, *SD* = 3.08 years), from the surrounding community participated in the experiment. Older participants were required to be 65 years of age or older. All participants gave informed consent in writing to participate in the study; the study and consent procedure were approved by the University of California, Riverside IRB. All observers were naïve concerning the experimental purpose and were paid for their participation in the experiment. All subjects were pre-screened for self-reported eye disease (e.g. macular degeneration, glaucoma, retinitis pigmentosa), neurological disorders (e.g. Alzheimers disease, Parkinson’s disease, stroke), as well as for any significant hearing loss. After passing this pre-screening participants were then assessed using an array of visual (log MAR acuity and contrast sensitivity) and cognitive (forward and backward digit span; WAIS matrix reasoning) tests (see Table 1). Participants were required to have a log minimum angle of resolution of 0.40 or better as measured using a standard 3 meter Original Series ETDRS Chart for far vision, as well as Sloan Two-Sided ETDRS Format Near Point Test to assess near vision. Log contrast sensitivity of 1.00 or better was also required for inclusion in the study and was measured using a Pelli-Robson Contrast Sensitivity Chart. Participants were also required to have digit-span scores no more than one standard deviation below the standards for cognitively normal individuals published by the Alzheimer’s Disease Center [25]. Visual and cognitive test data was lost for one older participant. The data for this participant was still included in the analyses, as this individual had minimum scores for all pre-tests for inclusion in the study (acuity, contrast sensitivity, and forward and backward digit span). Participants were allowed the use of hearing aids (only one participant required the use of a hearing aid), and hearing was tested using the pre-test described in the experimental methods. Any corrective lenses or contacts normally worn by the participants were also allowed during the experiment.

**Table 1:** Means and standard deviations of participant demographics and results from cognitive and perceptual tests.

	Younger		Older	
Variable	*M*	*SD*	*M*	*SD*
Age(years)^1^	23.17	4.26	71.45	3.08
Log Contrast Sensitivity^1^ ^,^ ^2^	1.46	0.13	1.35	0.07
Near LogMAR Acuity	0.11	0.18	0.21	0.14
Far LogMAR Acuity^1^	-0.02	0.12	0.13	0.16
Digit Span Forward	10.67	2.10	9.64	1.86
Digit Span Backwards	6.17	1.53	6.45	1.13
WAIS–Matrix Reasoning	20.58	3.37	14.82	5.44

Note:

^1^ Differences between age groups were significant as indicated by a two-tailed t-test (p < 0.05).

^2^ Contrast sensitivity measured using the Pelli Robson Test (Pelli, Robson & Wilkins, 1988).

### Apparatus

**S**timuli were presented on a 22” Viewsonic PF817 CRT monitor at a resolution of 1024x768 at 100Hz (non-interlaced) driven by an Alienware Area-51 ALX (Intel Core i7 960 processor, NVIDIA Geforce GTX 480 graphics card, Microsoft Windows 7 Service Pack 1). The background luminance of the display was 0.06 cd/m^2^. Sound was presented using five Dell A215 speakers. The experimental software was custom written in MATLAB (The Mathworks, Inc., version 7.8.0.347) and the Psychophysics Toolbox extensions were also used [26–28]. Calibration and measurements of the monitor were performed using a ColorCal2 colorimeter made by Cambridge Research Systems.

### Stimuli

Stimuli consisted of 1–2 flashes of a uniform white disc paired with 0–2 auditory beeps. The radius of the flashed disc was 0.75° in visual angle in size and was presented at the monitors maximum brightness level (127.97 cd/m^2^). The flashes had a duration of 10 milliseconds (ms) with a 70 ms inter-flash interval. Auditory beeps were 3500 Hz sine wave tones. The beeps were also 10 ms in duration with 70 ms inter-beep intervals, and were presented at 72 dB sound pressure level. All beeps had a 2 ms onset and offset ramp. On trials that included both flashes and beeps, the onset of the first beep began 20 ms before the onset of the first flash (the first beep occurring prior to the first flash were conditions that replicated the conditions in other studies examining the sound-induced flash illusion [17,18]). Previous research [17] has found no decrease in the strength of the illusion with temporal offsets of up to 70 ms.

### Experimental task and procedure

The monitor was viewed at a distance of 106 centimeters and head position was stabilized with the use of a chin rest. The only light source in the room during the experiment was the monitor. All stimuli were viewed binocularly.

At the beginning of the study all participants were required to pass a pre-test to ensure that they were able to discriminate the unimodal beeps and flashes that would be used in the study. The pre-test consisted of two blocks, the first assessed their ability to discriminate 1 or 2 beeps, and the second assessed their ability to discriminate 1 or 2 flashes. Beeps were presented from a single speaker 100cm from the participants that was placed directly below the front of the monitor. On each trial participants fixated a 0.5° crosshair, which was centered horizontally and presented 2.5° above the center of the screen. Participants then pressed any key to advance each trial. The stimuli were then presented which consisted of either flashes or beeps. During the flash pre-test block the flashes were presented 5° below the fixation crosshair. At the end of the trial participants were shown a blank response screen and entered the number of flashes or beeps perceived using the left arrow key to indicate one flash or beep and the right arrow key to indicate two flashes or beeps. Participants were given five blocks of 16 trials. The pre-test ended when they were able to get 13 of the 16 trials correct within a single block to be eligible for the study. No significant differences (p > 0.05) in the number of blocks taken to complete the pre-tests were observed between age groups for either block. This auditory pre-test was the only test of audition.

**Fig 1:**
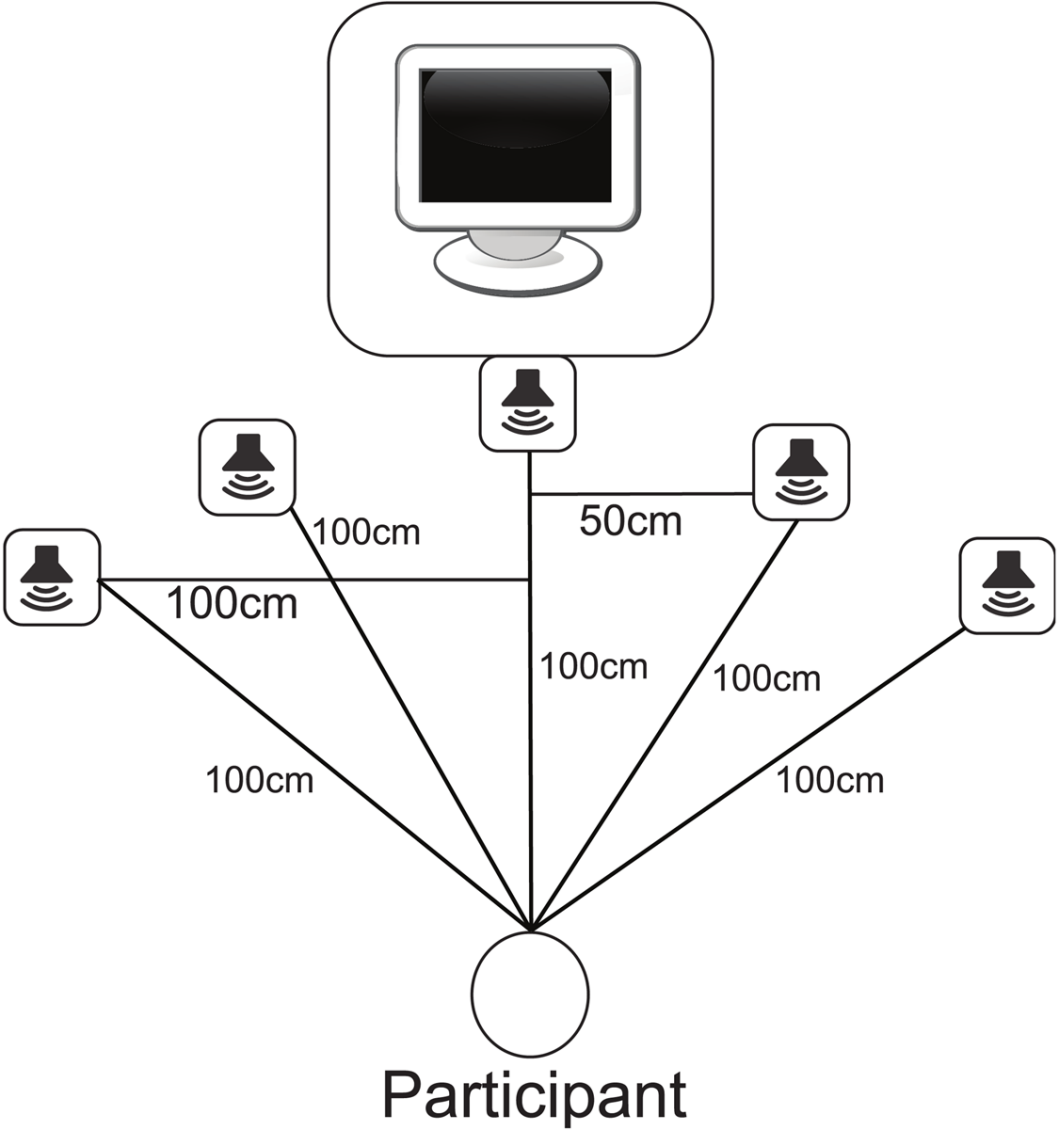
Experimental Layout. The experimental layout including the participant, monitor and speakers.

The experiment consisted of the two pre-tests and a single experimental block. The entire experiment took approximately 1 hour. The experimental block assessed participants’ ability to discriminate 1 to 2 visual flashes when paired with 0–2 beeps. The beeps came from one of five speakers placed 100 cm from the observer along a semi-circle (see Fig 1). This configuration was used to control for the effect of distance. All speakers were calibrated so that the beeps were presented at 72dB at the observer. Each possible combination of flashes, beeps and speaker displacement was presented 20 times during the experimental block, for a total of 560 trials. Presentation order was randomized for each participant. The task of the participant was to report the perceived number of flashes using the left and right arrow keys, with the left arrow key indicating one flash and the right arrow key indicating two. Participants were informed that the trials would frequently be accompanied by a series of beeps, and that while these beeps may be distracting to remember to respond only to the number of flashes presented. On each trial participants fixated on a 0.5° white crosshair presented 2.5° above the center of the screen that was presented for 250–1250 ms randomized on each trial. This variable onset timing was implemented to eliminate any strategy using the temporal length of each trial as a cue to the number of flashes presented. After the delay 1–2 visual flashes were presented 5° below the fixation cross. The crosshair then disappeared and text appeared on the screen instructing the participants to “Please enter ← if you saw one flash or → if you saw two flashes.” Participants then entered their response.

## Results

### Response Analysis

**Fig 2:**
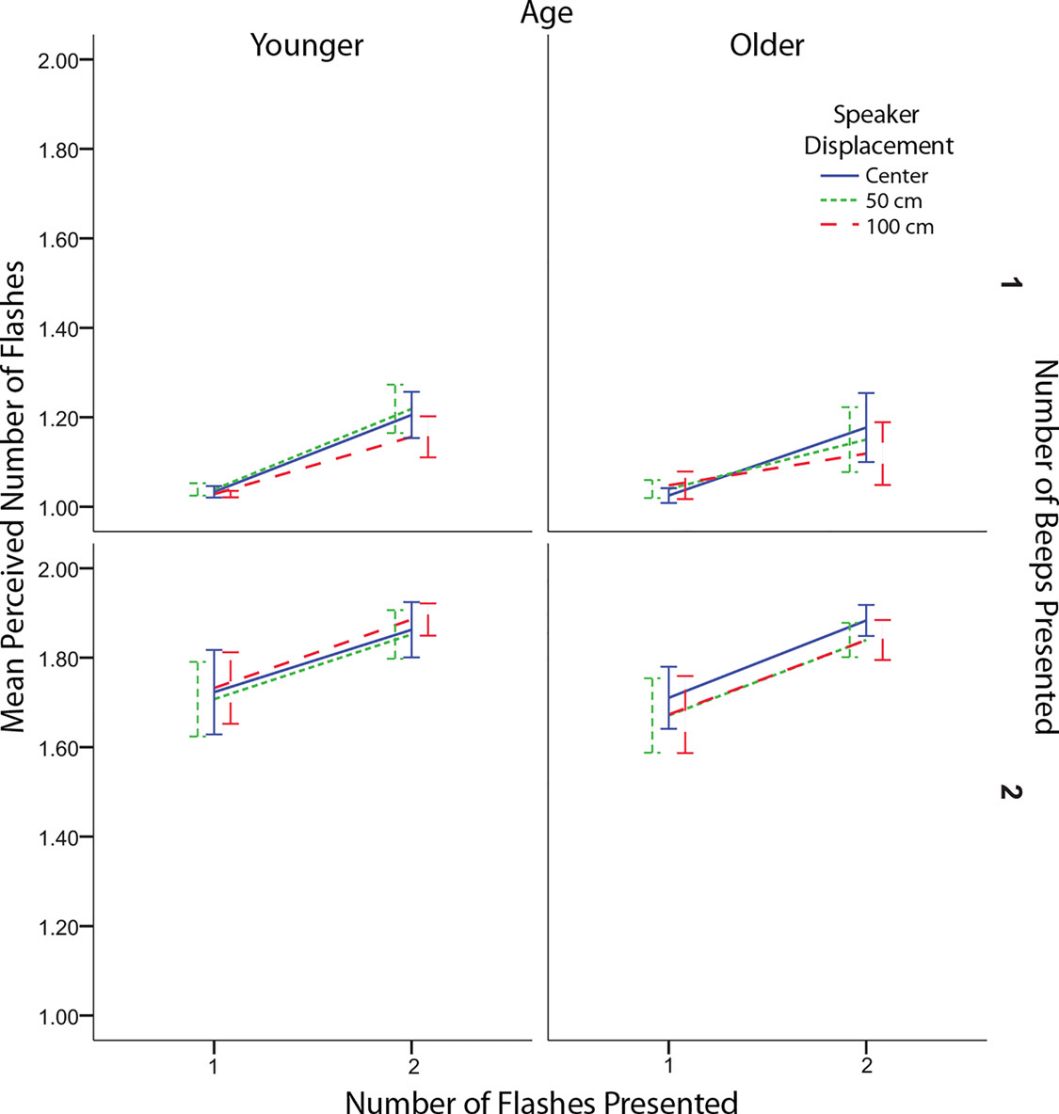
Effect of flashes, beeps, and speaker displacement on perceived number of flashes. Mean response as indicated by the number of flashes, number of beeps, age, and speaker displacement. Error bars indicate ± 1 standard error of the mean.

**Fig 3:**
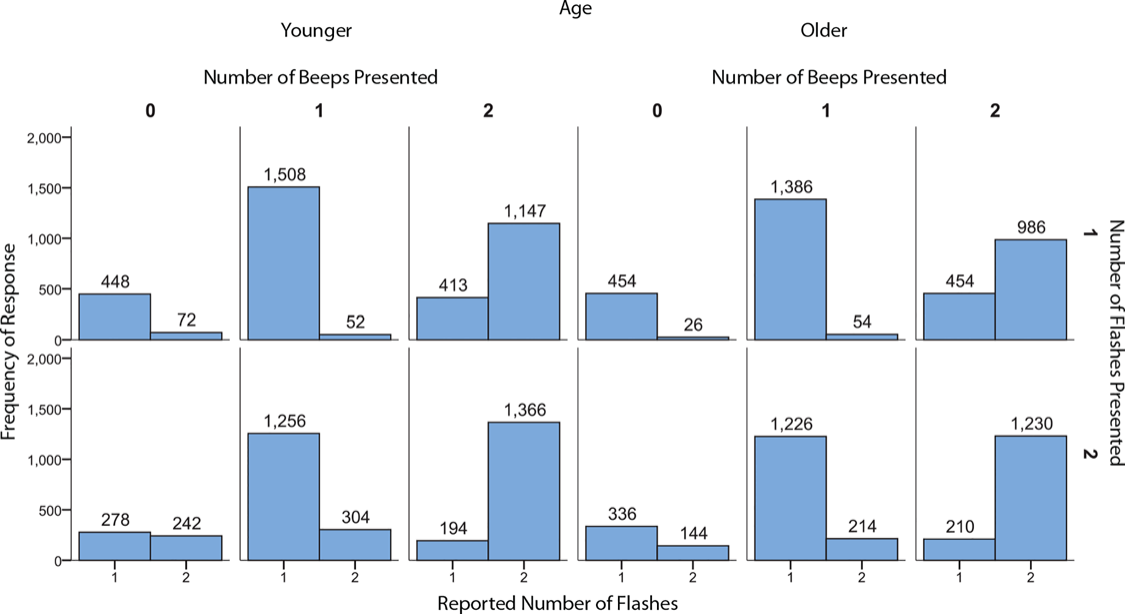
Response frequencies for all trial types. Response frequencies as indicated by age, number of flashes and number of beeps.

**Fig 4:**
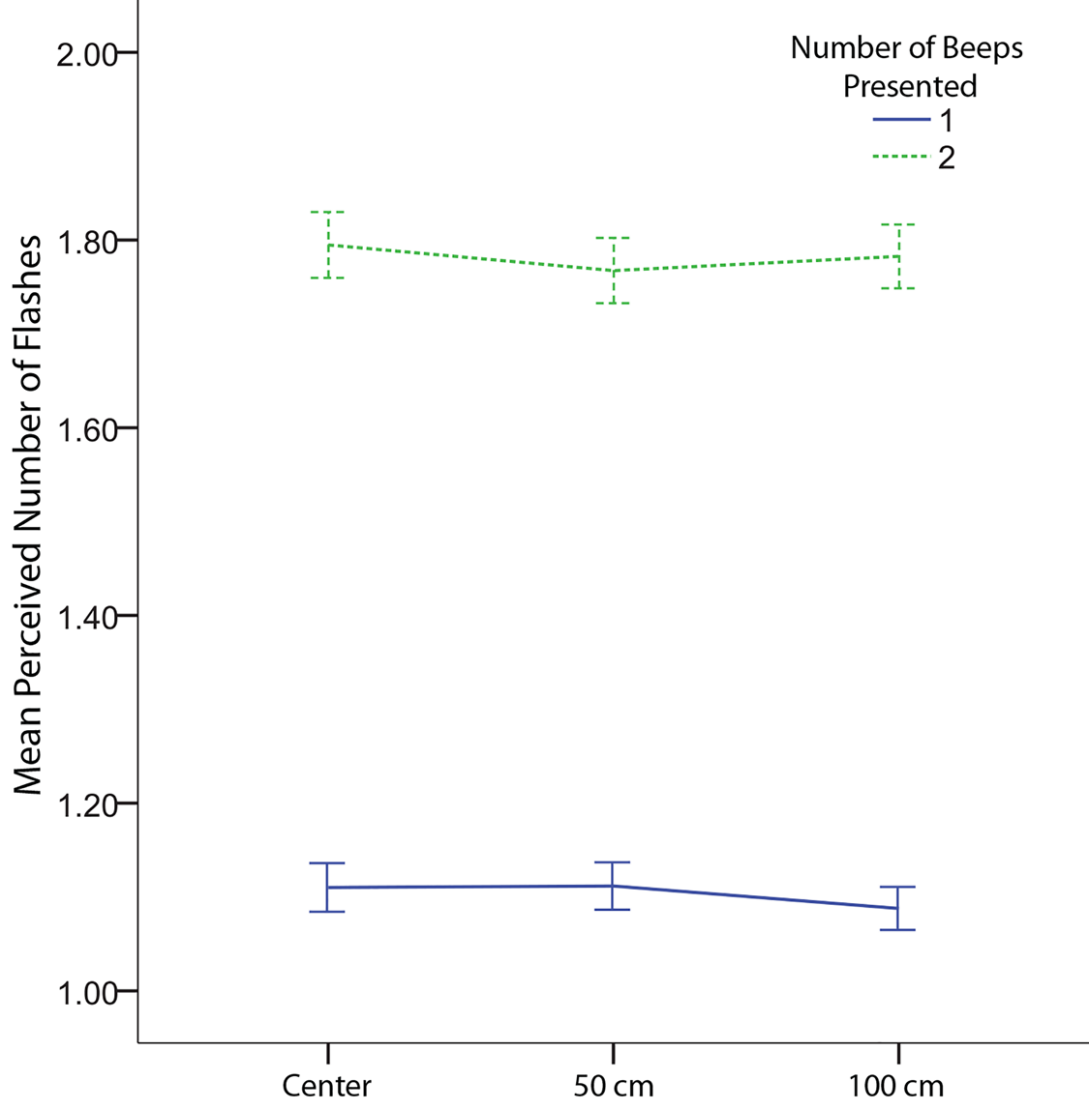
Interaction between beeps and speaker displacement. Mean response as indicated by speaker displacement and the number of beeps. Error bars indicated ± 1 standard error of the mean.

Response data was analyzed using an Age (2: Younger, Older) x Flashes (2: 1 flash, 2 flashes) x Beeps (2: 1 beep, 2 beeps) x Speaker Displacement (3: 0m, 0.5m, 1m) mixed analysis of variance. For any effects that violated sphericity we used Greenhouse-Geisser corrections. Original degrees of freedom are reported. As expected the number of flashes significantly influenced the perceived number of flashes (*F*(1,22) = 28.847, *p* < .001, see Fig 2). There was also a significant effect of beeps (*F*(1,22) = 308.02, *p* < .001, see Fig 2). The beeps showed a significant effect on the perceived number of flashes for both fusion, fission, and congruent trials (see Fig 3). There was greater accuracy for the two flash as compared to single flash trials when multisensory information was congruent. This effect was more pronounced for older as compared to younger adults because the older adults tended to underestimate the number of flashes in the unisensory two flash condition. A significant interaction between beeps and speaker displacement was also found (*F*(2,44) = 3.36, *p* = .044, see Fig 4). A simple effects analysis of speaker displacement split by number of beeps revealed that there was a significant effect of speaker displacement for trials with a single beep (*F*(2,32) = 4.268, *p* = .023). Localized analyses using paired two-tailed t-tests revealed a significant difference between the 0.5 meter speaker displacement and the 1 meter speaker displacement (*t*(16) = 2.90, *p* = 0.01), the difference between the 0 meter and 1 meter speaker displacements did not reach significance (*t*(16) = 2.05, *p* = 0.057). Surprisingly, the single beep had a stronger effect on responses with a 1 meter displacement as compared to 0.5 meters. At 0.5m there was a small, albeit non-significant shift towards a decrease in both the fission and fusion illusion. However, at 1m this shift showed a significant increase in the fusion illusion as compared to the 0.5m condition.

### Reaction Time Analysis

**Fig 5:**
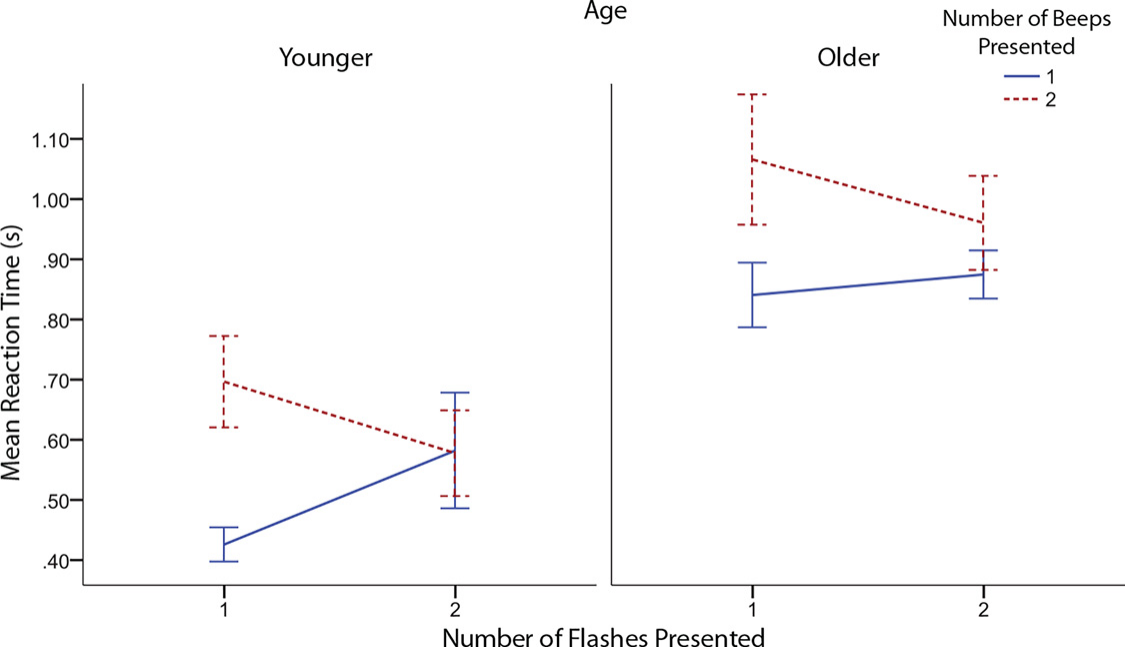
Reaction times depend on the presence of the illusion. Mean reaction times as indicated by age, number of flashes presented, and number of beeps presented. Error bars indicate ±1 standard error of the mean.

Reaction time data was analyzed using an Age (2: Younger, Older) x Flashes (2: 1 flash, 2 flashes) x Beeps (2: 1 beep, 2 beeps) x Speaker Displacement (3: 0m, 0.5m, 1m) mixed analysis of variance. For any effects that violated sphericity we used Greenhouse-Geisser corrections. Original degrees of freedom are reported. There was a significant main effect of beeps on reaction time (*F*(1,22) = 4.864, *p* = 0.038, see Fig 5). Reaction times for trials with two beeps were greater than those for one beep trials. However, this may have been partially determined by the interaction of flashes and beeps (*F*(1,22) = 5.012, *p* = 0.036). As depicted in Fig 5, the greater reaction for two beep conditions occurred primarily for the single flash as compared to the two flash condition. Interestingly, fusion illusion trials showed reaction times nearly 100ms longer than non-illusion congruent trials for both age groups as indicated by a simple effects analysis (*F*(1,22) = 5.613, *p* = 0.027). However, fission illusion trials showed no significant change in RT compared to congruent trials (*F*(1,22) = 3.253, *p* = 0.085), with nearly no RT difference in younger individuals, and a small but non-significant difference in older individuals (see Fig 5). Lastly, a significant effect of age was found (*F*(1,22) = 7.421, *p* = 0.012). Responses for older individuals were found to be significantly slower than the response times for younger individuals.

## Discussion

The present study examined age-related differences in the integration of spatially disparate multisensory stimuli using the sound-induced flash illusion. The results of the response data indicated no reliable difference in the sound-induced flash illusion due to increased spatial disparity for younger or older adults. This finding provides further evidence in support of previous research that found little or no impact of spatial disparity for audiovisual illusions in young adults [21]. In the present study, we examined spatial disparities that were greater than the disparities examined in previous research. This suggests that the lack of an effect of spatial disparity was not due to the smaller range of disparity examined. We also did not find a reliable difference in the effect of disparity between older or younger individuals. This suggests that the impact of spatially disparate visual and auditory information in the sound-induced flash illusion on multisensory perception may not differ with age. The results of these studies, considered together, suggest that the sound-induced flash illusion is resistant to spatial disparity [20].

Previous research on the ventriloquist effect has examined the role of spatially disparate visual and auditory information on a multisensory illusion [29]. The results of this research indicate that when the visual stimulus was strong, making visual localization easy, vision dominates spatially disparate auditory stimuli and the sound is perceived as spatially coincident with the visual stimulus location. However, as visual localization is made more difficult, the perceived location of the spatially disparate auditory stimuli is shifted less from its true source. In the present study, the visual flash was highly salient and easy to localize, which may have diminished the effect of spatial disparity for the auditory stimuli. This suggests that the failure to find an effect of spatial disparity might have been due to the use of a visual stimulus that was easy to spatially localize. An important issue for future research will be to examine whether degraded visual stimuli might impact the effect of spatial disparity and decrease the strength of the sound-induced flash illusion as would be expected due to response depression.

Both older and younger individuals had responses consistent with the fusion and fission illusions. This suggests a high degree of multimodal integration for both age groups. In addition, congruent flashes and beeps increased accuracy for both older and younger individuals, providing further evidence of a high degree of multimodal integration. These findings suggest that although audition and vision separately decline with age that the integration of these information sources when spatially disparate does not decline with age [12,30].

The reaction time results indicate an interesting increase in reaction time for fusion illusion trials for both older and younger individuals (see Fig 5). Reaction times for fusion illusion trials were nearly 100ms slower than those for congruent single flash trials. No significant difference in reaction times for fission illusion trials was found. Other research on the sound-induced flash illusion has found a larger increase in reaction times with adults in fission trials for both correct and incorrect responses [31]. However, for fusion trials an increase in reaction times is only seen when a correct answer is given. For incorrect responses, there was no significant increase in reaction times. These results suggest that there may be separate processes involved for the two illusion types, particularly when the illusion is perceived.

Another possibility is that there is more uncertainty in single flash trials in which participants may believe that they failed to observe the second flash. In contrast, this uncertainty for two flash trials would not occur if both flashes are successfully perceived. However, the frequency data suggests that this difference in uncertainty may not be present (see Fig 4). Both younger and older observers made more mistakes on flash only trials with two flashes as compared to single flash trials. This lends support to the hypothesis that the two forms of the illusion may be the result of different processes. Previous research examining the role of intensity on the sound-induced flash illusion in both younger and older individuals has also found evidence that the two illusions may be the result of two distinct processes [32], with a greater magnitude of the illusion for fusion as compared to fission trials. These differences could be due to a range of possibilities from low-level processing differences in sensory integration to higher-level issues such as a differential role of attention. An important issue for future research will be to examine this issue in detail.

Overall, the results of the present study suggest that the sound-induced flash illusion is not influenced by spatial disparity. Similar results for spatial disparity were obtained for both younger and older individuals, suggesting that the integration of spatially disparate stimuli does not decline with age. These results, considered with the results of previous research [8], suggest that although older adults show greater integration than younger adults with the sound induced flash illusion, this age dependent effect is not associated with age-related differences in in the integration of spatially disparate information. In addition, the results of the analysis of reaction times suggest that the two forms of the illusion may be due to separate processes. Future research will be needed to examine whether fusion and fission illusions are the result of two distinct processes.
